# HBEVOcc: Height-Aware Bird’s-Eye-View Representation for 3D Occupancy Prediction from Multi-Camera Images

**DOI:** 10.3390/s26030934

**Published:** 2026-02-01

**Authors:** Chuandong Lyu, Wenkai Li, Iman Yi Liao, Fengqian Ding, Han Liu, Hongchao Zhou

**Affiliations:** 1 School of Information Science and Engineering, Shandong University, Qingdao 266237, China; sdulcd@mail.sdu.edu.cn (C.L.); 202332680@mail.sdu.edu.cn (W.L.); 202220471@mail.sdu.edu.cn (F.D.); 202120419@mail.sdu.edu.cn (H.L.); 2 School of Computer Science, University of Nottingham Malaysia, Semenyih 43500, Malaysia; iman.liao@nottingham.edu.my

**Keywords:** autonomous driving, 3D occupancy prediction, multi-camera, BEV representation

## Abstract

Due to the ability to perceive fine-grained 3D scenes and recognize objects of arbitrary shapes, 3D occupancy prediction plays a crucial role in vision-centric autonomous driving and robotics. However, most existing methods rely on voxel-based methods, which inevitably demand a large amount of memory and computing resources. To address this challenge and facilitate more efficient 3D occupancy prediction, we propose HBEVOcc, a Bird’s-Eye-View based method for 3D scene representation with a novel height-aware deformable attention module, which can effectively leverage latent height information within BEV framework to compensate for lack of height dimension, significantly reducing computing resource consumption while enhancing the performance. Specifically, our method first extracts multi-camera image features and lifts these 2D features into 3D BEV occupancy features via explicit and implicit view transformations. The BEV features are then further processed by a BEV feature extraction network and height-aware deformable attention module, with the final 3D occupancy prediction results obtained through a prediction head. To further enhance voxel supervision along the height axis, we introduce a height-aware voxel loss with adaptive vertical weighting. Extensive experiments on the Occ3D-nuScenes and OpenOcc dataset demonstrate that HBEVOcc can achieve state-of-the-art results in terms of both mIoU and RayIoU metrics with less training memory (even when trained on 2080Ti).

## 1. Introduction

Accurate 3D perception is a crucial foundation for scene understanding and obstacle avoidance in autonomous driving and robotics. In recent years, vision-based 3D perception methods have garnered significant attention over LiDAR-based methods due to their lower cost, superior generalization, stability, as well as their ability to obtain richer color information. Some vision methods have demonstrated notable success in 3D perception tasks, such as 3D object detection [1,2,3,4,5,6], semantic map reconstruction [7,8], depth estimation [9,10,11], and motion prediction [12], etc.

Unlike the above visual 3D perception tasks, 3D occupancy prediction [13,14,15] takes multi-camera images as input and represents the real 3D world into voxels, estimating and predicting the semantic occupancy state of each voxel in the surrounding environment. 3D occupancy prediction provides more fine-grained 3D scene perception ability, capable of describing arbitrary complex shapes [16]. Moreover, 3D occupancy prediction models can identify both general objects and unusual obstacles, which is extremely important for scene understanding and reconstruction in autonomous driving and robotics. As an effective alternative to LiDAR-based perception, 3D occupancy prediction offers better assistance for downstream tasks and possesses a very broad development prospect.

Despite the aforementioned advantages, 3D occupancy prediction remains a highly challenging task that needs to achieve a balance of accuracy, robustness, and efficiency. Current 3D occupancy prediction methods mostly rely on voxel-based heavy 3D representation and processing, such as 3D convolutions and transformer operators [14,16,17]. These approaches lead to high computational cost and memory consumption, making them impractical for the actual perception requirements of autonomous driving and robotics. Recent works have aimed to address these issues through various optimizations. For instance, TPVFormer [15] uses tri-perspective view representations to reduce the amount of computation, and OctreeOcc [18] employs an octree structure to represent 3D scenes. However, these models still take up a large amount of memory during training.

Bird’s-Eye-View (BEV)-based methods have achieved remarkable success in 3D object detection with both accuracy and efficiency. Unlike voxel-based methods that explicitly model 3D spatial structure via voxels (leading to high memory and computational costs), BEV-based methods [1,2] project multi-view image features onto a 2D top-down plane, collapsing the height dimension into channel-wise features to enable efficient computation. However, in the task of 3D occupancy prediction, it is generally believed that BEV-based methods collapse the height information and are unable to effectively describe the fine-grained 3D scene details. Although some recent efforts have attempted to employ BEV representation for 3D occupancy prediction [19,20], they often fail to achieve satisfactory performance and do not achieve results comparable to voxel-based methods.

During the transformation from 2D image features to 3D occupancy features, there are two primary view transformations. One is the explicit view transformation (EVT) that performs forward projection based on the predicted depth map, and the other is the implicit view transformation (IVT) that conducts backward projection through cross-attention. The EVT can efficiently lift 2D image features to 3D space using the predicted depth map, but its drawback is that sparse LiDAR points limit the supervision of pixel-level depth prediction. On the other hand, IVT enables end-to-end transformation but suffers from inherent depth ambiguities.

To solve the above problems, we propose a BEV-based 3D occupancy prediction framework to achieve excellent results while reducing resource consumption. We adopt both explicit and implicit view transformations to take advantage of their strengths and compensate for their weaknesses simultaneously. To address the problem of the height information deficiency in BEV, we introduce a height-aware deformable attention module that can mine the potential latent height information, enabling interactions between features of the same and different heights. To complement this at the supervision level, we further introduce a height-aware voxel loss with adaptive height weighting to better guide the learning of sparsely distributed occupancy voxels.

Our contributions are summarized as follows:

- We design HBEVOcc, a framework leveraging BEV representation and a novel height-aware deformable attention module for 3D occupancy prediction. By effectively exploiting the latent height information embedded in BEV features, it addresses the absence of vertical dimensionality in BEV representations, resulting in a significant improvement in 3D occupancy prediction performance.
- Our proposed method learns 3D occupancy prediction from multi-camera images through both explicit and implicit view transformations. It enables the efficient fusion of explicit, implicit, and multi-scale BEV features, significantly reducing the memory usage of 3D occupancy prediction whilst maintaining high performance. To further improve height voxel supervision, we introduce a height-aware voxel loss with adaptive weighting along the height axis.
- Through extensive experiments on the Occ3D-nuScenes and OpenOcc dataset, we demonstrate that HBEVOcc outperforms existing methods in 3D occupancy prediction, achieving superior performance in this challenging task. Our results outperform not only BEV-based but also voxel-based methods, achieving a better trade-off between memory consumption and accuracy.

## 2. Related Work

### 2.1. Vision-Based 3D Occupancy Prediction

Recently, vision-based 3D occupancy prediction has attracted considerable attention in both academia and industry. PanoOcc [21] proposes a unified occupancy representation for camera-based 3D panoptic segmentation and occupancy prediction, aiming to integrate object detection and semantic segmentation into a single framework. It uses voxel queries to aggregate spatio-temporal information from multi-frame multi-view images via a coarse-to-fine scheme and introduces an occupancy sparsify module. RenderOcc [22] achieves 3D occupancy prediction using only 2D labels for supervision. SelfOcc [23] and OccNeRF [24] adopt a self-supervised approach for occupancy prediction, eliminating the dependence on occupancy labels. FB-OCC [25] enhances 3D occupancy prediction through forward–backward view transformation, integrating BEV and voxel representations, while employing depth and semantic pre-training. COTR [26] reconstructs a compact occupancy representation using a geometric encoder and a semantic decoder via the compact occupancy transformer. Nevertheless, it still relies on voxel-based modeling, which inherently incurs high GPU memory usage and computational costs, limiting scalability compared with BEV-based solutions. OctreeOcc [18] introduces a novel multi-granularity octree framework, which sparsifies the space and reduces the number of voxels. SAMOccNet [27] introduces the Segment Anything Model into occupancy prediction, enhancing fine-grained scene understanding through detailed visual feature extraction and fusion. OFMPNet [28] is an end-to-end model that jointly predicts future occupancy and motion flow using BEV inputs and a novel time-weighted loss. STCOcc [29] introduces a spatial–temporal cascade framework that explicitly utilizes the occupancy state to guide 3D feature refinement for improved scene understanding. Compared with these voxel-based methods, our approach avoids explicit voxelization and instead directly models height cues within BEV features. This design achieves effective height-aware occupancy prediction with significantly lower memory consumption and better scalability, making it more suitable for efficient deployment.

### 2.2. 3D Semantic Scene Completion

3D semantic scene completion (SSC) is most closely related to 3D occupancy prediction. It was first introduced in [30]. Monoscene [31] achieved 3D SSC using monocular image for the first time through 2D and 3D Unets, bridged by Feature Line of Sight Projection (FLoSP). VoxFormer [32] adopts a novel two-stage design, employing depth-based query proposals and a sparse voxel transformer with deformable cross-attention and self-attention to achieve 3D SSC. OccFormer [17] designs a dual-path transformer network and Mask2Former [33] to achieve semantic scene completion (SSC) and 3D occupancy prediction. OccDepth [34] exploits the implicit depth information in stereo images, using Stereo Soft Feature Assignment (STEREO-SFA) and Occupancy Aware Depth (OAD) modules to improve the effectiveness of 3D SSC. Symphonize [35] presents a novel paradigm that dynamically encodes instance-centric semantics, effectively mitigating geometric ambiguity through contextual scene reasoning.

### 2.3. BEV-Based 3D Scene Representation

BEV representations have been demonstrated to be a highly successful and effective approach in 3D object detection. BEV utilizes vectors to represent the features of BEV grids. Compared to voxel-based methods, BEV-based methods collapse the height dimension, thus improving the computational efficiency. BEVDet [1] projects image features into BEV features using predicted depth, achieving a good balance between accuracy and inference speed. BEVFormer [2] implements 3D object detection through a transformer and uses cross-attention and self-attention to complete the aggregation of spatial and temporal features. Recently, some works have also applied BEV methods to 3D occupancy prediction. FlashOcc [20] introduces a plug-and-play paradigm that replaces 3D convolutions with 2D convolutions, while using a channel-to-height prediction head to convert BEV features into 3D occupancy outputs. FastOcc [19] accelerates inference by collapsing voxel features into 2D BEV features, supplementing them with voxel features obtained through the interpolation of image features, while utilizing BEV semantic segmentation for supervision. Although the aforementioned methods utilize BEV for 3D occupancy prediction, there still remains a gap compared to voxel-based 3D occupancy prediction methods. DHD [36] introduces an explicit height prior into occupancy prediction by predicting height maps with LiDAR supervision and decoupling them into multiple height masks via the proposed Mask Guided Height Sampling (MGHS) module. These masks enable 2D features to be projected into separate 3D subspaces. This explicit height decoupling strategy improves the accuracy on Occ3D-nuScenes. However, DHD requires dense height labels and a relatively heavy architecture consisting of multiple dedicated modules (HeightNet, MGHS), which increases the model complexity and training overhead. In particular, projecting features into multiple height subspaces and aggregating them layer by layer incurs substantial GPU memory consumption, making DHD [36] less efficient compared to lightweight BEV-based approaches. In contrast, our method leverages height-aware deformable attention to implicitly mine latent vertical information already embedded in BEV features, without relying on external height labels or complex multi-stage subspace modeling. As a result, our framework achieves stronger efficiency–performance trade-offs: it improves height-aware representation while maintaining lightweight memory usage and architectural simplicity.

## 3. Proposed Method

### 3.1. Problem Formulation

Given a sequence of multi-camera image inputs, the aim of 3D occupancy prediction is to estimate the occupancy state and semantic category of each voxel in 3D space surrounding the ego-vehicle. Specifically, the input images are defined as $I_{i}^{t}\in R^{H_{i}\times W_{i}\times 3}$, where i∈{1,2,…,N} represents the *i*-th of *N* surround-view cameras, and t∈{T,T−1,…,T−τ} denotes the current timestamp at *T* with historical τ frames. Here, *H* and *W* indicate the height and width of the input images, respectively. Furthermore, the extrinsic parameters $\left\{\left[R_{i}|t_{i}\right]\right\}$ and intrinsic parameters $\left\{K_{i}\right\}$ of the camera used for different coordinate systems conversion and ego-motion in each frame are also known. The range of 3D space around the ego vehicle is $\left[X_{min},Y_{min},Z_{min},X_{max},Y_{max},Z_{max}\right]$, and the resolution of the voxel label for 3D occupancy prediction is [X,Y,Z] (e.g., [200,200,16] in Occ3D [14]), with each voxel representing a real-world size of $\left[\frac{X_{max}-X_{min}}{X},\frac{Y_{max}-Y_{min}}{Y},\frac{Z_{max}-Z_{min}}{Z}\right]$.

### 3.2. Overview

**Figure 1:**
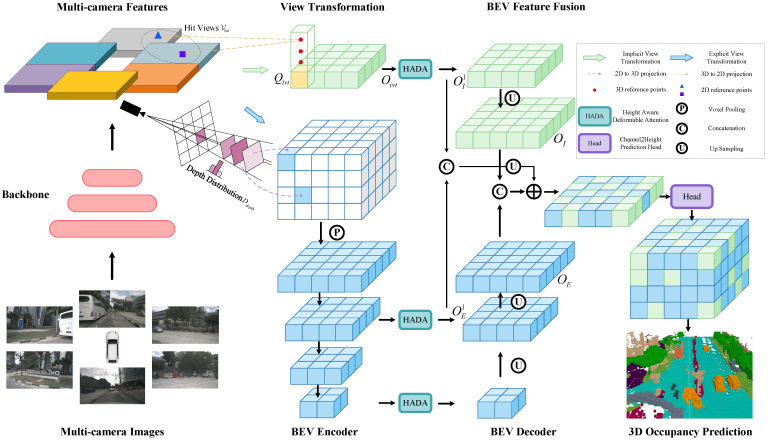
The overview of our proposed 3D occupancy prediction model (HBEVOcc). Firstly, we extract 2D features from multi-camera images using an image backbone network. Subsequently, we utilize EVT and IVT to lift 2D image features into 3D BEV space. We process BEV features by a BEV encoder and HADA module. Finally, we use a BEV decoder to recover spatial resolution, and then, we further fuse the explicit and implicit features to predict 3D occupancy through the prediction head.

Figure 1 shows the pipeline of our method. Given multi-camera images as input, we first extract features of images using a backbone network (e.g., ResNet-50 [37]); then, we lift the image features to 3D BEV space. After obtaining the initial features of BEV, a BEV encoder and height-aware deformable attention module are used to further refine the features, and then a BEV decoder progressively restores the spatial resolution. Finally, the explicit and implicit features are fused and fed into the prediction head to obtain the 3D occupancy results.

EVT and IVT are designed to address the inherent ambiguity and information loss when lifting multi-view image features into the BEV space by jointly modeling the explicit geometric projection and implicit learned occupancy queries.

### 3.3. Explicit View Transformation

We follow previous works such as FlashOcc [20] and BEVDet [4] to implement EVT, which lifts image features into 3D space using depth-based projections. After extracting features from a 2D backbone, we can obtain the multi-camera image features $F_{img}={\left\{F_{i}\in R^{C_{f}\times H_{f}\times W_{f}}\right\}}_{i=1}^{N}$. For EVT, the depth distribution $D_{depth}={\left\{D_{i}\in R^{D_{bin}\times H_{f}\times W_{f}}\right\}}_{i=1}^{N}$ can be predicted via a depth net, where $D_{bin}$ denotes the number of depth bins. The outer product $D_{depth}⊗F_{img}$ is applied to lift image features to pseudo-LiDAR points $P_{l}=R^{N\times D_{bin}\times C_{f}\times H_{f}\times W_{f}}$ in the camera coordinates. Then, $P_{l}$ is transformed to ego-coordinate and wrapped into a voxel grid with fixed resolution $\left[X,Y,Z_{e}\right]$ based on their 3D positions, where $Z_{e}$ is the height resolution along the *z* axis. Next, voxel pooling is performed to obtain the voxel feature $P_{l}^{1}\in R^{C_{f}\times X\times Y\times Z_{e}}$, which is subsequently permuted and reshaped into $P_{l}^{2}\in R^{(Z_{e}\times C_{f})\times X\times Y}$. Finally, a 2D convolutional preprocessing network is applied to produce the initial explicit BEV occupancy feature $O_{e}\in R^{C_{e}\times X\times Y}$.

### 3.4. Implicit View Transformation

For IVT, we adopt the query-based cross-attention strategy proposed in BEVFormer [2], where learnable BEV queries interact with multi-view image features via spatial cross-attention. In IVT, we only use the cross-attention component of BEVFormer [2], which projects 3D points into 2D image features to obtain the BEV features. As illustrated in Figure 1 (Implicit View Transformation), we predefine learnable parameters $Q_{ivt}\in R^{C_{ivt}\times \frac{X}{2}\times \frac{Y}{2}}$ as implicit BEV occupancy queries. Then, we obtain the corresponding implicit BEV occupancy feature $O_{ivt}\in R^{C_{ivt}\times \frac{X}{2}\times \frac{Y}{2}}$ using spatial cross-attention. The above process can be expressed as follows:

(1) $$O_{ivt}=\frac{1}{\left|V_{\mathrm{hit}}\right|}\sum_{i\in V_{\mathrm{hit}}}\mathrm{CA}\left(Q_{ivt},P_{i},F_{i}\right),$$

where $V_{\mathrm{hit}}$ is the hit views of 3D reference points in IVT, and CA(·) denotes the cross-attention. For each 3D point in $Q_{ivt}$, we use a project function $P_{i}$ to get the reference point on the *i*-th camera image.

### 3.5. Height-Aware Deformable Attention

**Figure 2:**
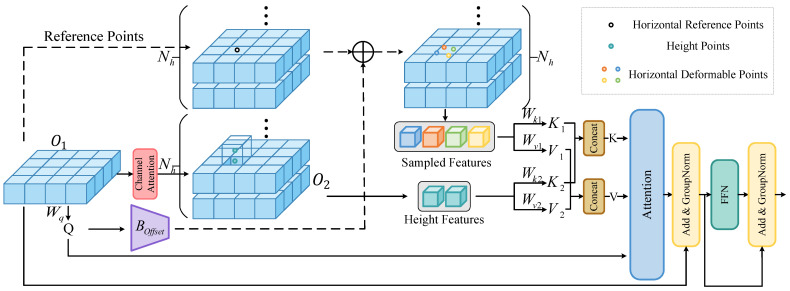
The architecture of height-aware deformable attention.

BEV-based methods collapse the height dimension into channel-wise features, leading to the loss of vertical spatial information and the inability to capture fine-grained 3D scene details. This deficiency limits their performance in 3D occupancy prediction, as height is critical for distinguishing objects and describing the spatial structure. To address the shortcoming of the lack of height information in BEV methods, we design a height-aware deformable attention (HADA) module, as shown in Figure 2. Inspired by [38], we also use deformable sampling points in the sampling process. After the initial BEV occupancy feature is passed through the BEV encoder, the BEV feature is $O_{1}\in R^{C_{1}\times X^{\prime }\times Y^{\prime }}$ (e.g., $O_{ivt}$), where $\left[X^{\prime },Y^{\prime }\right]$ is the resolution of the current BEV feature. A grid of reference points $p\in R^{\left(N_{h}\times 2\right)\times X^{\prime }\times Y^{\prime }}$ is predefined, where $N_{h}$ is the number of attentional height heads.

**Figure 3:**
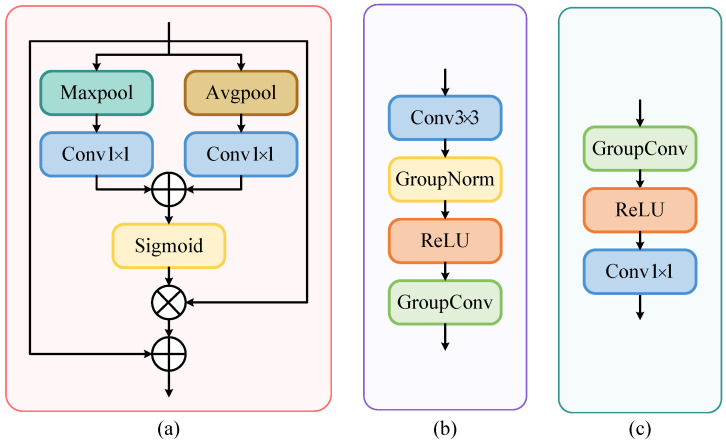
The architecture of each module in height-aware deformable attention. (**a**) Channel attention network. (**b**) Offset network. (**c**) Feedforward network.

We define the deformable sampling process as follows:

(2) $$\begin{array}{c} \Delta p=B_{offset}\left(O_{1}\right),O_{s}=Sample\left(W_{O_{1}}O_{1},p+\Delta p\right), \end{array}$$

where $\Delta p\in R^{\left(N_{h}\times p_{1}\times 2\right)\times X^{\prime }\times Y^{\prime }}$ is obtained from the 2D offset prediction network $B_{offset}$ shown in Figure 3b, and $p_{1}$ is the number of sampling points in the horizontal direction at each height. $W_{O_{1}}$ is a 2D convolutional network. Sample is the bilinear interpolation function, and $O_{s}\in R^{\left(N_{h}\times p_{1}\times C_{h}\right)\times X^{\prime }\times Y^{\prime }}$ is the sampled feature, where $C_{h}=C_{1}/N_{h}$ is the feature dimension per height head. $P_{sample}=p+\Delta p\in R^{\left(N_{h}\times p_{1}\times 2\right)\times X^{\prime }\times Y^{\prime }}$ is the corresponding sampling position. For this addition, the reference points *p* are broadcasted to match the dimensions of Δp by replicating each reference point $p_{1}$ times along the sampling point dimension; so, when we perform $P_{sample}$, *p* will broadcast to $\left(N_{h}\times p_{1}\times 2\right)\times X^{\prime }\times Y^{\prime }$. The query, key, and value features are then computed as

(3) $$Q=W_{q}O_{1},K_{1}=W_{K1}O_{s},V_{1}=W_{v1}O_{s}.$$

After using the 2D convolutional network $W_{q}$ on $O_{1}$, $Q\in R^{\left(N_{h}\times C_{h}\right)\times X^{\prime }\times Y^{\prime }}$ is obtained. We use GroupConv in $W_{K1}$ and $W_{v1}$; then, we obtain $K_{1}\in R^{\left(N_{h}\times p_{1}\times C_{h}\right)\times X^{\prime }\times Y^{\prime }}$, $V_{1}\in R^{\left(N_{h}\times p_{1}\times C_{h}\right)\times X^{\prime }\times Y^{\prime }}$.

In the height direction, we process $O_{1}$ using channel attention shown in Figure 3a,

(4) $$\begin{array}{c} O_{2}=Channel\;Attention\left(O_{1}\right),K_{2}=W_{K2}O_{2},V_{2}=W_{v2}O_{2}, \end{array}$$

where $O_{2}\in R^{C_{1}\times X^{\prime }\times Y^{\prime }}$, $K_{2}\in R^{\left(N_{h}\times p_{2}\times C_{h}\right)\times X^{\prime }\times Y^{\prime }}$, $V_{2}\in R^{\left(N_{h}\times p_{2}\times C_{h}\right)\times X^{\prime }\times Y^{\prime }}$, and $p_{2}$ is the number of points in the height direction. Then, we compute attention by the following equation:

(5) $$\begin{array}{c} K=Concat\left(K_{1},K_{2}\right),V=Concat\left(V_{1},V_{2}\right),\\ O_{A}=Attention\left(Q,K,V\right)=Softmax\left(\frac{QK^{T}}{\sqrt{C_{h}}}\right)V, \end{array}$$

where the shapes of *K* and *V* are both $\left(N_{h}\times \left(p_{1}+p_{2}\right)\times C_{h}\right)\times X^{\prime }\times Y^{\prime }$. When we calculate the attention $O_{A}$, *Q* only calculates with nearby points; so, *Q* will be reshaped to $\left(N_{h}\times 1\times C_{h}\right)\times X^{\prime }\times Y^{\prime }$, $K^{T}\in R^{\left(N_{h}\times C_{h}\times \left(p_{1}+p_{2}\right)\right)\times X^{\prime }\times Y^{\prime }}$, $QK^{T}\in R^{\left(N_{h}\times 1\times \left(p_{1}+p_{2}\right)\right)\times X^{\prime }\times Y^{\prime }}$, and $O_{A}\in R^{\left(N_{h}\times 1\times C_{h}\right)\times X^{\prime }\times Y^{\prime }}$. After the $O_{A}$ is processed by feedforward network (FFN), the shape of the final output of HADA is $C_{1}\times X^{\prime }\times Y^{\prime }$. As can be seen from Figure 2 and Figure 3c, we use GroupConv and GroupNorm in FFN, which can achieve different processing of features at different heights.

### 3.6. 3D Occupancy Prediction Head

After the BEV processing network, the explicit and implicit BEV occupancy features are denoted as $O_{E}\in R^{C_{E}\times X\times Y}$ and $O_{I}\in R^{C_{I}\times X\times Y}$, respectively. We upsample the low-resolution features $O_{E}^{1}\in R^{C_{E}^{1}\times \frac{X}{2}\times \frac{Y}{2}}$ and $O_{I}^{1}=HADA\left(O_{ivt}\right)\in R^{C_{I}^{1}\times \frac{X}{2}\times \frac{Y}{2}}$ shown in Figure 1 and then fuse them with $O_{E}$ and $O_{I}$ to obtain their fused feature $O_{F}\in R^{C_{F}\times X\times Y}$. Specifically, the explicit and implicit features are concatenated along the channel dimension, and the upsampled low-resolution fused features are added to the high-resolution fused features, followed by a convolution layer for feature fusion. The above process can be expressed as follows:

(6) $$\begin{array}{c} O_{F}=Conv\left(Concat\left(O_{E},O_{I}\right)+Upsample\left(Concat\left(O_{E}^{1},O_{I}^{1}\right)\right)\right). \end{array}$$

Like FlashOcc [20], we also employ Channel2Height prediction head, and we obtain $O_{F1}\in R^{(Z\times C)\times X\times Y}$ after applying a 1×1 convolution on $O_{F}$. Finally, we permute and reshape $O_{F1}$ to $O\in R^{X\times Y\times Z\times C}$ to get the final 3D occupancy prediction output.

### 3.7. Height-Aware Voxel Loss

In 3D occupancy prediction, voxels are unevenly distributed along the height axis (Z-axis): voxels near the ground (e.g., 0–2 m) are densely occupied by objects like vehicles and pedestrians, while voxels at higher altitudes (e.g., 2–5.4 m) are sparsely occupied. Conventional loss functions treat all voxels equally, leading to biased supervision—models prioritize learning from dense low-height voxels and underperform on sparse high-height voxels, reducing the overall prediction accuracy.

**Figure 4:**
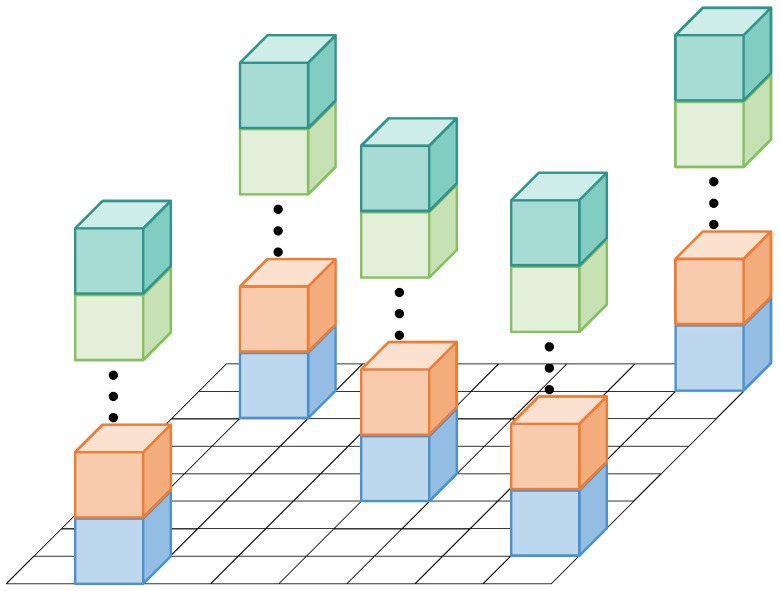
Height voxel loss. Different colors represent randomly selected voxels at different heights.

To address this issue, we propose a height-aware voxel loss (HAVL), which enhances voxel supervision along the height axis by introducing adaptive height-dependent weighting. Specifically, we randomly sample a set of positions in the XY plane and compute the loss over all height levels at those positions as shown in Figure 4. This sampling strategy reduces the computational cost while ensuring that vertical occupancy patterns are jointly optimized.

For each height, a weight is assigned based on the number of occupied voxels at that height. Heights with fewer occupied voxels are assigned larger weights. To penalize low-confidence errors more strongly, we apply the loss to log function. This design increases the gradient magnitude when the predicted probability approaches zero, which is particularly important for sparse occupancy voxels. We adopt Smooth L1 to prevent excessively large gradients caused by extreme log-probability values. The final loss is formulated as

(7) $$L_{HAV}=\mathrm{SmoothL}1\left(\sum_{z=1}^{Z}\sum_{(x,y)\in S}w_{z}\cdot log\left(p_{c}^{\left(x,y,z\right)}\right),0\right),$$

where $w_{z}=w_{\max}\cdot {\left(\frac{w_{\min}}{w_{\max}}\right)}^{\frac{N_{z}}{N_{\max}}}$ is the height-aware weight at height *z*, $N_{z}$ is the number of occupied voxels at height *i*, and $N_{\max}$ is the maximum among all $N_{z}$. This exponential scheme emphasizes sparse heights and ensures smooth weighting without hard thresholds. (x,y)∈S denotes randomly sampled positions in the XY plane, and $p_{c}^{\left(x,y,z\right)}$ is the predicted probability of ground-truth class *c* at voxel (x,y,z).

### 3.8. Model Optimization

In the model training stage, we use the cross-entropy loss $L_{ce}$, depth loss $L_{depth}$ supervised by sparse LiDAR points, lovasz-softmax loss $L_{lovasz}$ from [39], affinity loss $L_{sem}$ and $L_{geo}$ from MonoScene [31], and our proposed height-aware voxel loss $L_{HAV}$ to optimize our model. So, the total loss function used for our model optimization can be defined as follows:

(8) $$\begin{array}{c} L_{total}=\lambda_{depth}L_{depth}+\lambda_{ce}L_{ce}+\lambda_{lovasz}L_{lovasz}\\ +\lambda_{sem}L_{sem}+\lambda_{geo}L_{geo}+\lambda_{HAV}L_{HAV}, \end{array}$$

where λ is the weight of each loss, and we set $\lambda_{depth}=0.15$, $\lambda_{ce}=\lambda_{lovasz}=1$ following [4,20,36,40] in our experiments. The weight for $\lambda_{sem}=\lambda_{geo}=\lambda_{HAV}=0.1$ is chosen to ensure numerical consistency with the existing loss terms for stable training.

## 4. Experiments

### 4.1. Dataset

We conduct experiments on two large scale 3D occupancy prediction benchmarks: Occ3D-nuScenes [14] and OpenOcc [41]. Each dataset comprises 700, 150, and 150 scenes in the training, validation, and testing sets, respectively, and each scene is 20 s in duration and 2 HZ in frequency. Each frame consists of 6 surround-view camera images. 3D occupancy labels have a spatial range of [−40 m, −40 m, −1 m, 40 m, 40 m, 40 m, 5.4 m] across the *X*, *Y*, and *Z* axes, a voxel resolution of [200, 200, 16], and a size of each voxel of [0.4 m, 0.4 m, 0.4 m]. Each voxel of Occ3D-nuScenes has 18 categories (1 “other” class and 1 “free” class), and the labels provide a visible mask for the camera. OpenOcc annotates each voxel with 17 categories (1 “free” class) and provides per-voxel motion flow prediction.

### 4.2. Experimental Settings

#### Implementation Details

For explicit BEV features, our initial resolution is set to 200×200, and the encoder follows the design of FlashOcc [20]. HADA is applied at resolutions of 100×100 and 25×25, where the BEV feature dimensions are set to 128/512 for single-frame input and 160/640 when temporal history frames are used. Accordingly, the explicit BEV feature $O_{e}$ has a shape of 200×200×64 or 200×200×80, depending on the presence of a temporal input. For EVT, the voxel grid resolution along the *z* axis, denoted as $Z_{e}$, is set to 8 for single frame or 1 history frame input and reduced to 1 for multiple historical frames inputs to save computation. For implicit BEV features, the initial resolution is 100×100, with feature dimensions of 128 and 160 for single frame and temporal inputs, respectively. The resulting BEV feature $O_{i}$ has a shape of 100×100×128 or 100×100×160, followed by an upsampling operation and a two-layer convolution. HADA is applied at a resolution of 100×100. In HBEVOcc, both explicit and implicit BEV outputs, $O_{E}$ and $O_{I}$, are unified to a channel dimension of 256, and the fused BEV feature $O_{F}$ has a final dimension $C_{F}$ of 512. Under the setting without history frames, our approach does not rely on LiDAR-based depth supervision. We also construct a fast version, HBEVOcc-Fast, where an explicit and implicit BEV feature is added at the 100×100 resolution, and HADA is applied solely at 25×25. In this case, the final fused feature $C_{F}$ is reduced to 256. For temporal fusion in HBEVOcc and HBEVOcc-Fast, we adopt Stereo4D and Depth4D used in [4,20,36,40]. Specifically, when computing the BEV features of historical frames, gradients are disabled to reduce the memory usage. After obtaining 3D features from both the current and historical frames, we concatenate them after a lightweight preprocessing network and then feed the concatenated features into the BEV encoder.

### 4.3. Evaluation Metrics

For 3D semantic occupancy prediction, we use mIoU as the evaluation metric on Occ3D-nuScenes [14]. In addition, we also use the RayIoU proposed in SparseOcc [42] as the evaluation metric both on Occ3D and OpenOcc [41], which is defined as TP only if the class are consistent and the L1 distance between the predicted depth and the true depth is less than a certain threshold. We use the mean absolute velocity error (mAVE) to evaluate the scene flow prediction across defined categories (e.g., pedestrian, bus) on OpenOcc.

#### Training

During training, we adopt the AdamW [43] optimizer with a learning rate of 2 × 10^−4^ and weight decay of 0.01, using a linear warming up in the first 200 iterations. Models with a ResNet-50 backbone are trained on 8 RTX2080Ti GPUs (11 G memory) and those with a SwinB backbone are trained on 4 RTX4090 GPUs (24 G memory), both with a batch size of 2.

### 4.4. Main Results

#### 4.4.1. 3D Occupancy Prediction Results on Occ3D-nuScenes

**Figure 5:**
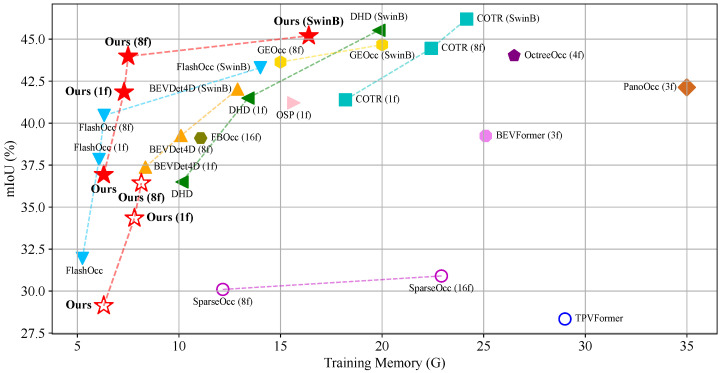
Comparison of mIoU and training memory of various 3D occupancy prediction methods on the Occ3D-nuScenes dataset. Different colors and shapes represent different methods. “1f” and “8f” mean fusing temporal information from 1 and 8 history frames using ResNet-50 backbone. The solid and hollow shapes represent whether the camera mask is used for training or not.

**Figure 6:**
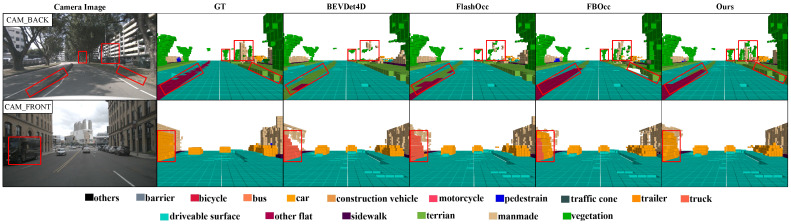
Qualitative visualization comparison results on the Occ3D-nuScenes dataset. The first column is a camera image, the second column is the ground truth, and the rest are the 3D occupancy prediction results of BEVDet4D, FlashOcc, FBOcc, and our method HBEVOcc.

We report the quantitative results and qualitative visualizations of the 3D semantic occupancy prediction results on the Occ3D-nuScenes dataset. In Table 1, we report in detail the comparison results of our HBEVOcc and other existing state-of-the-art methods on mIoU and each semantic class. Our method consistently achieves the best performance, regardless of whether camera masks or history frames are used. In Figure 5, we visualize the training memory and performance comparison between HBEVOcc and other methods. Our method uses less memory but achieves better performance. In Figure 6, we visualize the results of our model and the state-of-the-art methods. Our method can predict the occupancy semantic classes more accurately compared to SOTA.

In Table 2, we also report in detail the comparison results of our HBEVOcc, HBEVOcc-Fast, and other existing methods on RayIoU and mIoU. Testing GPU is RTX 4090 refers to that we test the model according to its official code. Regardless of whether a camera mask is used or not, our method achieves state-of-the-art RayIoU while ensuring fast speed.

#### 4.4.2. 3D Occupancy Prediction Results on OpenOcc

In Table 3, we report the results of our HBEVOcc and other existing methods on RayIoU and mAVE. Our model achieves better occupancy and flow prediction results while consuming less memory (only 7.5 GB).

### 4.5. Ablation Study

**Figure 7:**
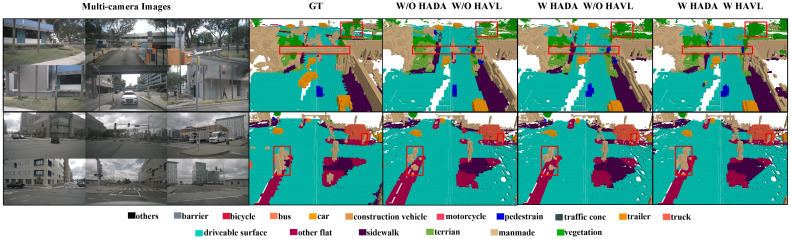
Qualitative visualization results for ablation study on the Occ3D-nuScenes dataset. The leftmost part shows surround-view camera image inputs, the second part shows the ground truth, and the last three parts visualize the 3D occupancy predictions under three settings: (1) without HADA and HAVL, (2) with HADA only, and (3) with both HADA and HAVL.

To verify the effectiveness of our proposed method and module, we perform ablation experiments on the Occ3D and OpenOcc dataset. For a fair comparison, we retrain FlashOcc [20] with the additional loss function, including the lovasz-softmax loss $L_{lovasz}$ from [39] and the affinity loss $L_{sem}$ and $L_{geo}$ from MonoScene [31] and treat this enhanced model as our baseline. As shown in Table 4, incorporating both EVT and IVT results in a 0.98% improvement in mIoU compared to using EVT alone, demonstrating the benefits of their combination. Meanwhile, using HADA has a 1.08% higher mIoU than not using it, underscoring the effectiveness of HADA. In Figure 7, we visualize the 3D occupancy prediction results under three settings. It can be seen that our proposed HADA and HAVL enhance the geometric structure and semantic coherence of the 3D occupancy results, thereby improving scene understanding ability. Table 5 further shows that without using a camera mask, HADA can still improve RayIoU and mIoU to some extent, and HAVL can improve the mIoU by 1.7% without increasing the memory usage. Table 6 demonstrates the improvement effects of HADA and HAVL on OpenOcc. Due to the long training time, we use the image resolution of 256 × 704 and the image backbone ResNet-50 in the ablation experiments, and no history frame is used to reduce the training time.

As shown in Table 7, concatenation achieves the best mIoU performance among all evaluated fusion methods, outperforming additive fusion and gated fusion. In Table 8, we present the impact of different horizontal and height points in HADA on mIoU. The best performance is achieved when the horizontal point is 4 and the height point is 2. To investigate the impact of the voxel grid resolution along the *z* axis (height $Z_{e}$) setting under different numbers of historical frames, we conduct experiments on Occ3D dataset in Table 9. When no historical frame or only a single historical frame is used, adopting $Z_{e}=8$ leads to better performance compared with $Z_{e}=1$. However, when the number of historical frames increases to 4 or 8, $Z_{e}=1$ consistently outperforms $Z_{e}=8$. The temporal fusion of multiple historical frames provides additional geometric cues, which compensates for the potential loss of vertical information caused by a smaller z-dimension.

We first investigate the influence of the number of height levels involved in HAVL. As shown in Table 10, increasing the number of height levels generally improves the performance, with the best result achieved when 16 height levels are used. This indicates that sufficiently fine-grained height supervision is beneficial for learning sparse occupancy patterns along the vertical axis, while too coarse height partitioning limits its effectiveness. We further analyze the effect of the number of sampled spatial positions in the XY plane. Table 11 shows that sampling 4000 positions achieves the best performance, while overly dense sampling (e.g., 20,000 or 40,000) does not bring further improvement. This suggests that HAVL does not rely on exhaustive voxel supervision, and a moderate number of sampled positions is sufficient to provide stable and effective height-aware gradients. Table 12 shows that, in HAVL, the best performance is achieved when using our proposed height-aware weights.

**Figure 8:**
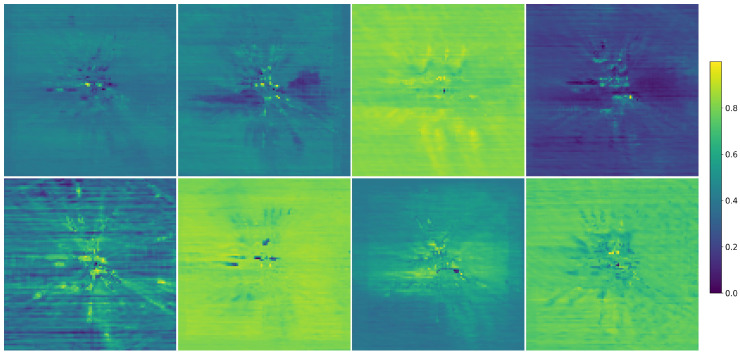
Visualization of the HADA attention maps at eight different height levels. Different colors in the heatmaps indicate varying intensities of the attention features.

Table 13 shows the effect of different history frames in temporal fusion on RayIoU, and it can be seen that long-term sequences lead to a significant improvement in the results. To evaluate the capability of HADA in mining and exploiting height information, we visualize in Figure 8 the heatmaps of attention $O_{A}$ features at eight different height levels. As shown in Figure 8, the regions emphasized by HADA vary across different heights, indicating that the model attends to distinct spatial features, depending on the vertical dimension. This clearly demonstrates the effectiveness of HADA in capturing and leveraging height-aware representations. To quantify how HADA’s height setting impacts performance across different vertical ranges, we conduct additional ablation experiments by varying the number of height levels in HADA (2, 4, 8, 16). Table 14 presents the results: the model achieves the best mIoU of 36.76% when using 8 height levels. This result confirms that 8 height levels strike an optimal balance between capturing vertical details and computational efficiency. To further verify the effectiveness of our proposed HADA, we applied it to other methods. For BEV-based methods, HADA is directly applied to the BEV features. For voxel-based methods, we first transform the voxel features into BEV features by collapsing the *z* axis into the channel dimension and applying a 2D convolutional network. HADA is then applied to the transformed BEV features, which are subsequently mapped back into the voxel representation and fused with the original voxel features through addition. As can be seen in Table 15, both BEV-based and voxel-based models achieve a significant improvement in mIoU with only a limited increase in memory usage. Here, mIoU* denotes the performance of the original methods; to ensure the fairness of the experiment, we retrain these methods to obtain the mIoU.

## 5. Discussion

As shown in Table 1, Table 2 and Table 3, our method consistently outperforms previous BEV-based and voxel-based approaches in terms of the occupancy prediction accuracy, while maintaining competitive inference efficiency. Compared with voxel-based methods, our approach avoids explicit 3D voxel computation and benefits from the compact BEV representation, leading to reduced inference memory consumption and favorable runtime performance. Although the proposed HADA module introduces additional computation, its cost is moderate due to the localized and sparse sampling strategy, making the overall complexity suitable for real-time or near-real-time deployment. Despite its effectiveness, our method has several limitations. First, extremely complex scenes with a large number of small or highly detailed objects may still pose challenges due to the inherent resolution limits of the BEV grid. Second, the current framework assumes relatively accurate camera calibration, and calibration errors may negatively impact the lifting process. Finally, like most vision-based methods, our approach may be affected by challenging environmental conditions such as low illumination, adverse weather, or sensor noise. In future work, incorporating temporal information or robustness-oriented data augmentation could further enhance performance under such conditions.

## 6. Conclusions

In this paper, we present HBEVOcc, a 3D occupancy prediction method based on BEV representation. To enhance the perception and understanding of 3D scenes, we employ both explicit and implicit view transformations to obtain BEV features. Our proposed HADA module and HAVL can effectively utilize the latent height information, addressing the challenge of missing height in BEV and significantly improving model performance. Our method achieves superior 3D occupancy prediction results while also reducing the training memory. Extensive experiments on the Occ3D-nuScenes and OpenOcc dataset demonstrate that HBEVOcc outperforms existing methods in both mIoU and RayIoU metrics, which proves the effectiveness of our proposed method.

## Figures and Tables

**Table 1:** 3D Occupancy prediction results (mIoU) on Occ3D-nuScenes dataset. All results are from official papers or codes. The best results are marked in bold.

Method	Mask	History Frame	Backbone	Image Size	mIoU (%) ↑	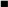 others	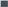 barrier	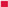 bicycle	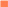 bus	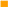 car	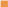 cons. veh.	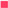 motorcycle	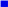 pedestrian	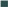 traffic cone	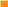 trailer	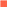 truck	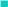 drive. surf.	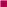 other flat	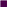 sidewalk	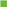 terrain	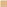 manmade	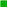 vegetation
MonoScene [31]	✗	✗	ResNet-101	900 × 1600	6.06	1.75	7.23	4.26	4.93	9.38	5.67	3.98	3.01	5.90	4.45	7.17	14.91	6.32	7.92	7.43	1.01	7.65
OccFormer [17]	✗	✗	ResNet-101	900 × 1600	21.93	5.94	30.29	12.32	34.40	39.17	14.44	16.45	17.22	9.27	13.90	26.36	50.99	30.96	34.66	22.73	6.76	6.97
TPVFormer [15]	✗	✗	ResNet-101	900 × 1600	28.34	6.67	39.20	14.24	41.54	**46.98**	**19.21**	22.64	17.87	14.54	**30.20**	**35.51**	56.18	33.65	35.69	31.61	19.97	16.12
CTF-Occ [14]	✗	✗	ResNet-101	900 × 1600	28.53	**8.09**	**39.33**	**20.56**	38.29	42.24	16.93	**24.52**	**22.72**	**21.05**	22.98	31.11	53.33	33.84	37.98	33.23	20.79	18.00
**HBEVOcc (ours)**	✗	✗	ResNet-50	256 × 704	**29.13**	6.48	37.65	18.05	**38.66**	42.56	18.45	21.72	19.94	18.13	21.43	30.03	**62.66**	**34.33**	**39.94**	**37.39**	**24.01**	**23.73**
BEVFormer [2]	✗	3	ResNet-101	900 × 1600	23.67	5.03	38.79	9.98	34.41	41.09	13.24	16.50	18.15	17.83	18.66	27.70	48.95	27.73	29.08	25.38	15.41	14.46
BEVStereo [3]	✗	1	ResNet-101	900 × 1600	24.51	5.73	38.41	7.88	38.70	41.20	17.56	17.33	14.69	10.31	16.84	29.62	54.08	28.92	32.68	26.54	18.74	17.49
SparseOcc [42]	✗	16	ResNet-50	256 × 704	30.9	10.6	39.2	20.2	32.9	43.3	19.4	23.8	23.4	29.3	21.4	29.3	67.7	36.3	44.6	40.9	22.0	21.9
**HBEVOcc (ours)**	✗	1	ResNet-50	256 × 704	**34.34**	**10.51**	**45.41**	**24.32**	**41.10**	**47.65**	**23.79**	**26.59**	**24.68**	**27.29**	**27.88**	**34.58**	**65.02**	**35.38**	**42.83**	**40.49**	**35.03**	**31.23**
**HBEVOcc (ours)**	✗	8	ResNet-50	256 × 704	**36.43**	**12.69**	**49.13**	**27.13**	**41.18**	**49.32**	**23.47**	**29.71**	**27.36**	**32.12**	**29.03**	**36.43**	**67.01**	**37.16**	**44.71**	**41.71**	**37.69**	**33.48**
BEVDetOcc [4]	✔	✗	ResNet-50	256 × 704	31.64	6.65	36.97	8.33	38.69	44.46	15.21	13.67	16.39	15.27	27.11	31.04	78.70	36.45	48.27	51.68	36.82	32.09
FlashOcc [20]	✔	✗	ResNet-50	256 × 704	31.95	6.21	39.57	11.27	36.32	43.95	16.25	14.73	16.89	15.76	28.56	30.91	78.16	37.52	47.42	51.35	36.79	31.42
DHD-S [36]	✔	✗	ResNet-50	256 × 704	36.50	10.59	43.21	23.02	**40.61**	47.31	21.68	23.25	23.85	23.40	**31.75**	34.15	**80.16**	41.30	49.95	**54.07**	**38.73**	33.51
**HBEVOcc (ours)**	✔	✗	ResNet-50	256 × 704	**36.93**	**11.00**	**44.07**	**23.83**	40.46	**48.9**	**22.29**	**24.49**	**25.80**	**25.80**	29.19	**34.24**	79.82	**41.32**	**50.33**	53.56	38.54	**34.16**
BEVDet4D [4]	✔	1	ResNet-50	256 × 704	36.01	8.22	44.21	10.34	42.08	49.63	23.37	17.41	21.49	19.70	31.33	37.09	80.13	37.37	50.41	54.29	45.56	39.59
FlashOcc [20]	✔	1	ResNet-50	256 × 704	37.84	9.08	46.32	17.71	42.7	50.64	23.72	20.13	22.34	24.09	30.26	37.39	81.68	40.13	52.34	56.46	47.69	40.6
OSP [44]	✔	1	ResNet-101	900 × 1600	41.21	10.95	49.0	27.68	50.24	**55.99**	22.96	**31.02**	**30.91**	30.25	35.60	**41.23**	82.09	42.59	51.9	55.1	44.82	38.17
COTR (BEVDet4D) [26]	✔	1	ResNet-50	256 × 704	41.39	12.20	48.51	**29.08**	44.66	53.33	27.01	29.19	28.91	30.98	35.03	39.50	81.83	42.53	53.71	56.86	48.18	**42.09**
DHD-M [36]	✔	1	ResNet-50	256 × 704	41.49	12.72	48.68	26.31	43.22	52.92	27.33	28.49	28.52	30.02	**35.81**	40.24	**83.12**	**44.67**	**54.71**	**57.69**	**48.87**	42.09
**HBEVOcc (ours)**	✔	1	ResNet-50	256 × 704	**41.84**	**13.28**	**49.96**	28.88	**45.76**	53.77	**28.19**	29.68	29.20	**32.38**	34.77	40.27	82.28	44.00	53.60	56.78	47.23	41.20
FBOCC [25]	✔	16	ResNet-50	256 × 704	39.11	13.57	44.74	27.01	45.41	49.1	25.15	26.33	27.86	27.79	32.28	36.75	80.07	42.76	51.18	55.13	42.19	37.53
FastOcc [19]	✔	16	ResNet-101	640 × 1600	39.21	12.06	43.53	28.04	44.80	52.16	22.96	29.14	29.68	26.98	30.81	38.44	82.04	41.93	51.92	53.71	41.04	35.49
BEVFormer [2]	✔	3	ResNet-101	900 × 1600	39.24	10.13	47.91	24.90	47.57	54.52	20.23	28.85	28.02	25.73	33.03	38.56	81.98	40.65	50.93	53.02	43.86	37.15
BEVDet4D [4]	✔	8	ResNet-50	384 × 704	39.26	9.33	47.05	19.23	41.47	52.21	27.19	22.23	23.32	21.58	35.77	38.94	82.48	40.42	53.75	57.71	49.94	**45.76**
CVT-Occ [45]	✔	6	ResNet-101	900 × 1600	40.34	9.45	**49.46**	23.57	49.18	55.63	23.1	27.85	28.88	29.07	34.97	40.98	81.44	40.92	51.37	54.25	45.94	39.71
ViewFormer [46]	✔	3	ResNet-50	256 × 704	41.85	12.94	50.11	27.97	44.61	52.85	22.38	29.62	28.01	29.28	35.18	39.40	**84.71**	**49.39**	**57.44**	**59.69**	47.37	40.56
PanoOcc [21]	✔	3	ResNet-101	900 × 1600	42.13	11.67	50.48	**29.64**	**49.44**	55.52	23.29	33.26	30.55	30.99	34.43	42.57	83.31	44.23	54.40	56.04	45.94	40.40
GEOcc [47]	✔	8	ResNet-50	256 × 704	43.64	14.29	51.27	31.11	46.13	55.09	**29.12**	30.46	30.99	35.47	35.2	41.82	84.0	47.0	55.52	59.5	50.03	44.82
**HBEVOcc (ours)**	✔	8	ResNet-50	256 × 704	**43.98**	**14.38**	**52.89**	30.65	46.29	**55.84**	29.00	**33.29**	**32.15**	**36.42**	**37.12**	**41.99**	82.86	45.48	54.91	58.96	**50.77**	44.6
BEVDet4D [4]	✔	1	Swin-B	512 × 1408	42.02	12.15	49.63	25.1	52.02	54.46	27.87	27.99	28.94	27.23	36.43	42.22	82.31	43.29	54.46	57.9	48.61	43.55
FlashOcc [20]	✔	1	Swin-B	512 × 1408	43.52	13.42	51.07	27.68	51.57	56.22	27.27	29.98	29.93	29.80	37.77	43.52	**83.81**	46.55	56.15	**59.56**	**50.84**	**44.67**
GEOcc [47]	✔	8	Swin-B	512 × 1408	44.67	14.02	51.4	33.08	52.08	56.72	**30.04**	33.54	32.34	35.83	**39.34**	44.18	83.49	46.77	55.72	58.94	48.85	43.0
**HBEVOcc (ours)**	✔	1	Swin-B	512 × 1408	**45.20**	**15.03**	**52.51**	**33.66**	**52.98**	**56.93**	29.03	**34.54**	**33.41**	**35.83**	38.58	**44.29**	83.79	**47.42**	**56.24**	59.33	50.53	44.30

**Table 2:** 3D Occupancy prediction results (RayIoU) on Occ3D-nuScenes dataset. All results are from official papers or codes. The best results are marked in bold.

Method	Mask	History Frames	Backbone	Input Size	Epoch	RayIoU (%) ↑	RayIoU_1m, 2m, 4m_ ↑			mIoU (%) ↑	FPS↑	Training Mem (G) ↓	Inference Mem (G) ↓	Training GPU	Testing GPU
SimpleOccupancy [48]	✔	✗	ResNet-101	336 × 672	12	22.5	17.0	22.7	27.9	31.8	9.7	-	-	A100	A100
BEVFormer [2]	✔	3	ResNet-101	900 × 1600	24	32.4	26.1	32.9	38.0	39.2	3.0	25.1	6.7	A100	A100
BEVDet4D [4]	✔	1	ResNet-50	256 × 704	90	29.6	23.6	30.0	35.1	36.1	2.6	8.4	4.7	A100	A100
BEVDet4D [4]	✔	8	ResNet-50	384 × 704	90	32.6	26.6	33.1	38.2	39.3	0.8	10.1	6.4	A100	A100
FBOcc [25]	✔	16	ResNet-50	256 × 704	90	33.5	26.7	34.1	39.7	39.1	10.3	11.1	5.5	A100	A100
**HBEVOcc-Fast(ours)**	✔	1	ResNet-50	256 × 704	24	**31.4**	24.8	31.8	37.6	**39.1**	**18.9**	6.4	2.7	RTX 2080Ti	RTX 4090
**HBEVOcc-Fast(ours)**	✔	8	ResNet-50	256 × 704	24	**33.4**	26.9	33.8	39.4	**41.2**	**14.6**	6.9	2.8	RTX 2080Ti	RTX 4090
**HBEVOcc (ours)**	✔	1	ResNet-50	256 × 704	24	**33.4**	26.9	33.8	39.4	**41.8**	8.2	7.3	3.0	RTX 2080Ti	RTX 4090
**HBEVOcc (ours)**	✔	8	ResNet-50	256 × 704	24	**34.9**	**28.6**	**35.4**	**40.8**	**44.0**	5.4	7.5	3.1	RTX 2080Ti	RTX 4090
SparseOcc [42]	✗	8	ResNet-50	256 × 704	24	34.0	28.0	34.7	39.4	30.1	17.1	12.2	5.4	A100	RTX 4090
SparseOcc [42]	✗	16	ResNet-50	256 × 704	24	35.1	29.1	35.8	40.3	30.6	14.1	22.9	6.9	A100	RTX 4090
SparseOcc [42]	✗	16	ResNet-50	256 × 704	48	36.1	30.2	36.8	41.2	30.9	14.1	22.9	6.9	A100	RTX 4090
Panoptic-FlashOcc [40]	✗	1	ResNet-50	256 × 704	24	36.0	30.1	36.8	41.1	29.6	**39.4**	6.1	2.2	A100	RTX 4090
Panoptic-FlashOcc [40]	✗	8	ResNet-50	256 × 704	24	38.5	32.8	39.3	43.4	31.5	**20.4**	6.3	2.4	A100	RTX 4090
GSD-Occ [49]	✗	16	ResNet-50	256 × 704	24	38.9	-	-	-	-	20.0	-	4.8	A100	A100
**HBEVOcc-Fast (ours)**	✗	1	ResNet-50	256 × 704	24	**37.1**	30.9	37.9	42.5	**31.7**	18.9	6.4	2.7	RTX 2080Ti	RTX 4090
**HBEVOcc-Fast (ours)**	✗	8	ResNet-50	256 × 704	24	**39.6**	33.4	40.3	45.0	**34.0**	14.6	6.9	2.8	RTX 2080Ti	RTX 4090
**HBEVOcc-Fast (ours)**	✗	8	ResNet-50	256 × 704	48	**40.1**	34.2	40.8	45.3	**34.2**	14.6	6.9	2.8	RTX 2080Ti	RTX 4090
**HBEVOcc (ours)**	✗	1	ResNet-50	256 × 704	24	**39.2**	33.3	40.0	44.4	**34.3**	8.2	7.3	3.0	RTX 2080Ti	RTX 4090
**HBEVOcc (ours)**	✗	8	ResNet-50	256 × 704	24	**41.0**	35.9	41.8	45.5	**36.4**	5.4	7.5	3.1	RTX 2080Ti	RTX 4090
**HBEVOcc (ours)**	✗	8	ResNet-50	256 × 704	48	**41.5**	**36.1**	**42.2**	**46.3**	**36.5**	5.4	7.5	3.1	RTX 2080Ti	RTX 4090

**Table 3:** 3D occupancy prediction results (RayIoU and mAVE) on OpenOcc. C and L denote camera and Lidar supervision. The best results are marked in bold.

Method	Sup.	Backbone	Input Size	History Frames	Epoch	RayIoU (%) ↑	mAVE ↓	FPS ↑	Training Mem (G) ↓	Inference Mem (G) ↓	Training GPU	Testing GPU
OccNeRF-C [24]	C	R101	900 × 1600	-	-	21.6	1.53	-	-	-	-	-
OccNeRF-L [24]	L	R101	900 × 160	-	-	31.7	1.59	-	-	-	-	-
RenderOcc [22]	L	R101	900 × 160	6	12	36.7	1.63	-	-	-	-	-
Let Occ Flow [50]	C+L	R101	512 × 1408	2	16	40.5	1.45	-	-	-	-	-
OccNet [41]	3D	R101	900 × 160	3	24	39.7	1.61	-	-	-	-	-
BEVFormer [2]	3D	R50	900 × 160	3	24	28.1	1.12	3.0	26.0	6.7	A100	A100
FB-Occ [25]	3D	R50	256 × 704	16	90	32.3	0.83	10.3	11.1	5.5	A100	A100
SparseOcc [42]	3D	R50	256 × 704	8	48	33.4	0.87	17.1	15.8	5.4	A100	RTX 4090
STCOcc [29]	3D	R50	256 × 704	16	48	40.8	0.44	4.7	10.0	5.6	RTX 4090	RTX 4090
**HBEVOcc (ours)**	3D	R50	256 × 704	1	24	**39.4**	**0.52**	8.2	7.3	3.0	RTX 2080Ti	RTX 4090
**HBEVOcc (ours)**	3D	R50	256 × 704	8	24	**40.8**	**0.41**	5.4	7.5	3.1	RTX 2080Ti	RTX 4090
**HBEVOcc (ours)**	3D	R50	256 × 704	8	48	**41.4**	**0.39**	5.4	7.5	3.1	RTX 2080Ti	RTX 4090

**Table 4:** mIoU of HBEVOcc for ablation study on Occ3D. The best results are marked in bold.

Baseline	EVT	IVT	HADA	HAVL	mIoU (%) ↑	Training Mem (G) ↓	Inference Mem (G) ↓	Params (M)	GFLOPs
✔					34.34	4.8	2.3	44.9	253.1
✔			✔		35.02	5.1	2.3	50.1	259.1
✔				✔	34.43	4.8	2.3	44.9	253.1
✔			✔	✔	35.13	5.1	2.3	50.1	259.1
	✔				34.70	5.0	2.3	45.8	280.3
		✔			34.40	4.1	2.2	28.1	148.7
	✔	✔			35.68	5.3	2.5	50.7	384.5
	✔	✔	✔		36.76	6.3	2.6	56.2	393.6
	✔	✔	✔	✔	**36.93**	6.3	2.6	56.2	393.6

**Table 5:** RayIoU of HBEVOcc for ablation study on Occ3D. The best results are marked in bold.

Baseline	EVT	IVT	HADA	HAVL	RayIoU (%) ↑	mIoU (%) ↑	Training Mem (G) ↓	Inference Mem (G) ↓	Params (M)	GFLOPs
✔					32.12	25.51	4.8	2.3	44.9	253.1
✔			✔		32.28	25.90	5.1	2.3	50.1	259.1
✔				✔	32.25	27.27	4.8	2.3	44.9	253.1
✔			✔	✔	33.11	27.81	5.1	2.3	50.1	259.1
	✔				32.58	25.97	5.0	2.3	45.8	280.3
		✔			32.51	26.24	4.1	2.2	28.1	148.7
	✔	✔			33.63	26.97	5.3	2.5	50.7	384.5
	✔	✔	✔		34.01	27.43	6.3	2.6	56.2	393.6
	✔	✔	✔	✔	**34.30**	**29.13**	6.3	2.6	56.2	393.6

**Table 6:** RayIoU of HBEVOcc for ablation study on OpenOcc. The best results are marked in bold.

Baeline	EVT	IVT	HADA	HAVL	RayIoU (%) ↑	mAVE ↓	Training Mem (G) ↓	Inference Mem (G) ↓	Params (M)	GFLOPs
✔					31.95	1.20	4.8	2.3	45.1	261.6
✔			✔		32.01	1.12	5.1	2.3	50.3	267.7
✔				✔	32.83	1.81	4.8	2.3	45.1	261.6
✔			✔	✔	32.92	1.75	5.1	2.3	50.3	267.7
	✔				32.16	1.02	5.0	2.3	46.0	285.9
		✔			32.09	1.37	4.1	2.2	28.2	154.1
	✔	✔			33.19	1.14	5.3	2.5	51.0	395.1
	✔	✔	✔		33.67	**1.01**	6.3	2.6	56.5	404.2
	✔	✔	✔	✔	**34.27**	1.03	6.3	2.6	56.5	404.2

**Table 7:** Different fusion methods of EVT and IVT. The best results are marked in bold.

Fusion Methods	mIoU (%) ↑	Training Mem (G) ↓	Inference Mem (G) ↓	Params (M)	GFLOPs
Concat	**35.68**	5.3	2.5	50.7	384.5
Add	35.42	5.2	2.3	47.7	297.6
Gated Fusion	35.58	5.4	2.4	48.1	308.8

**Table 8:** Effect of different number of horizontal and height points. The best results are marked in bold.

History Frames	Horizontal Points	Height Points	mIoU (%) ↑	Training Mem (G) ↓	Inference Mem (G) ↓	Params (M)	GFLOPs
✗	2	1	36.67	6.2	2.6	55.6	392.3
✗	2	2	36.68	6.2	2.6	56.2	393.3
✗	4	1	36.70	6.3	2.6	55.6	392.6
✗	4	2	**36.76**	6.3	2.6	56.2	393.6
1	2	1	41.36	7.2	3.0	73.4	640.8
1	2	2	41.63	7.2	3.0	74.3	642.3
1	4	1	41.56	7.3	3.0	73.4	641.2
1	4	2	**41.64**	7.3	3.0	74.3	642.7

**Table 9:** Effect of different heights $Z_{e}$ in different history frames on Occ3D. The best results are marked in bold.

History Frames	Height $Z_{e}$	mIoU (%) ↑	Training Mem (G) ↓	Inference Mem (G) ↓	Params (M)	GFLOPs
✗	1	36.78	6.2	2.6	55.6	370.0
	8	**36.93**	6.3	2.6	56.2	393.6
1	1	41.48	7.3	3.0	73.5	578.2
	8	**41.84**	7.3	3.0	74.3	642.7
4	1	**42.94**	7.3	3.1	74.1	946.8
	8	42.21	7.5	3.1	75.0	1131.2
8	1	**43.98**	7.5	3.1	75.0	1450.7
	8	42.58	7.6	3.1	75.9	1782.7

**Table 10:** Effect of different heights in height-aware voxel loss. The best results are marked in bold.

HAVL Height	mIoU (%) ↑	Training Mem (G) ↓	Inference Mem (G) ↓	Params (M)	GFLOPs
2	35.80	5.3	2.5	50.7	384.5
4	35.86	5.3	2.5	50.7	384.5
8	35.82	5.3	2.5	50.7	384.5
16	**36.00**	5.3	2.5	50.7	384.5

**Table 11:** Effect of different number of sampled positions in height-aware voxel loss. The best results are marked in bold.

Sampled Positions	mIoU (%) ↑	Training Mem (G) ↓	Inference Mem (G) ↓	Params (M)	GFLOPs
2000	35.87	5.3	2.5	50.7	384.5
4000	**36.00**	5.3	2.5	50.7	384.5
20,000	35.71	5.3	2.5	50.7	384.5
40,000	35.81	5.3	2.5	50.7	384.5

**Table 12:** Effect of different weight strategies in height-aware voxel loss. The best results are marked in bold.

HAVL	mIoU (%) ↑	Training Mem (G) ↓	Inference Mem (G) ↓	Params (M)	GFLOPs
✗	35.68	5.3	2.5	50.7	384.5
Fixed Weight $w_{z}=1$	35.89	5.3	2.5	50.7	384.5
Fixed Weight $w_{z}=2$	35.80	5.3	2.5	50.7	384.5
Fixed Weight $w_{z}=3$	35.84	5.3	2.5	50.7	384.5
Height-aware Weight	**36.00**	5.3	2.5	50.7	384.5

**Table 13:** Effect of different numbers of history frames on Occ3D and OpenOcc. The best results are marked in bold.

Dataset	History Frames	RayIoU (%) ↑	Training Mem (G) ↓	Inference Mem (G) ↓	Params (M)	GFLOPs
Occ3D-nuScenes	1	39.21	7.3	3.0	74.3	642.7
	4	40.50	7.3	3.1	74.1	946.8
	8	**41.05**	7.5	3.1	75.0	1450.7
OpenOcc	1	39.43	7.3	3.0	74.6	653.3
	4	40.02	7.3	3.1	74.3	957.4
	8	**40.78**	7.5	3.1	75.3	1461.3

**Table 14:** Effect of different heights in height-aware deformable attention. The best results are marked in bold.

HADA Height	mIoU (%) ↑	Training Mem (G) ↓	Inference Mem (G) ↓	Params (M)	GFLOPs
2	36.66	6.3	2.6	56.8	395.6
4	36.59	6.3	2.6	56.4	394.2
8	**36.76**	6.3	2.6	56.2	393.6
16	36.70	6.3	2.6	56.1	393.2

**Table 15:** Effectivenss of our proposed HADA with different methods. Here, mIoU* denotes the performance of the original methods; to ensure the fairness of the experiment, we retrain these methods to obtain the mIoU.

Methods	Representation	History Frames	mIoU * ↑	mIoU ↑	mIoU ↑ (+HADA)	ΔmIoU ↑	ΔMem (G) ↓
DHD-S [36]	BEV	✗	36.50	36.51	36.99	+0.48	+0.94
DHD-M [36]	BEV	1	41.49	40.74	41.36	+0.62	+1.09
FlashOcc:M2 [20]	BEV	✗	32.08	32.62	33.54	+0.92	+0.29
FlashOcc-4D-Stereo:M2 [20]	BEV	1	37.84	38.80	39.73	+0.93	+0.31
BEVDet4D [4]	Voxel	1	36.01	37.40	38.35	+0.95	+0.98
FBOcc [25]	BEV and Voxel	16	39.11	40.21	40.65	+0.44	+0.66

## Data Availability

The data presented in this study are openly available in HBEVOcc Available online: https://github.com/lvchuandong/HBEVOcc (accessed on 20 January 2026).
